# Cardiovascular magnetic resonance and echocardiographic findings of a large thrombosed intramyocardial dissecting hematoma: a case report and a brief review of literature

**DOI:** 10.1259/bjrcr.20200028

**Published:** 2020-06-03

**Authors:** Amal Abdelsattar Sakrana, Shadha A. Ahmed Alzubaidi, Abdulhameed Mohmmed Shahat, Abeer Sabri Mahmoud, Hesham Abdo Naeim

**Affiliations:** 1 Madina Cardiac Center, 23411 AL Madinah Al munawwrah, Khaled Bin Al Waleed Road, AL Madinah Al munawwrah, Saudi Arabia; 2 Department of Diagnostic and Interventional radiology, Mansoura University Hospital, 35112 12 El-Gomhoreya street, Mansoura, Egypt

## Abstract

Intramyocardial dissecting hematoma (IMDH) is an uncommon fatal complication after acute myocardial infarction. It is usually under identified. Transthoracic echocardiography is the first-line modality that can detect IMDH. Cardiac magnetic resonance could confirm the diagnosis. In this paper, we reported a unique partially thrombosed large left ventricle IMDH that mimics thrombosed true aneurysm aiming to highlight the supporting diagnostic transthoracic echocardiography and cardiac magnetic resonance criteria of IMDH.

## Introduction

Intramyocardial dissecting hematoma (IMDH) is a rare prognostically poor form of cardiac rupture with a haemorrhagic dissection through the myocardium.^1^ It occurs as a sequel of acute myocardial infarction due to therupture of the intramyocardial vessels or due to a sudden increase in the coronary perfusion pressure.^2^ Pathologically, it is formed of intramyocardial blood with intact endocardial and epicardial integrity.^3^ IMDH can involve the free or septal wall of the left ventricle (LV) or the right ventricle free wall.^4^ The most commonly used method for diagnosis is echocardiography. However, cardiovascular magnetic resonance (CMR) imaging is very beneficial to confirm the diagnosis. We will present transthoracic echocardiography (TTE) and CMR findings of an LV anterior wall and apex IMDH of our patient, who was treated conservatively.

## Case presentation

A 60-years-old male patient admitted to our centre as a case of anterior wall myocardial infarction with pulmonary oedema. He had a history of ischaemic heart disease, no history of hypertension, and he is not diabetic.

**Figure 1.**
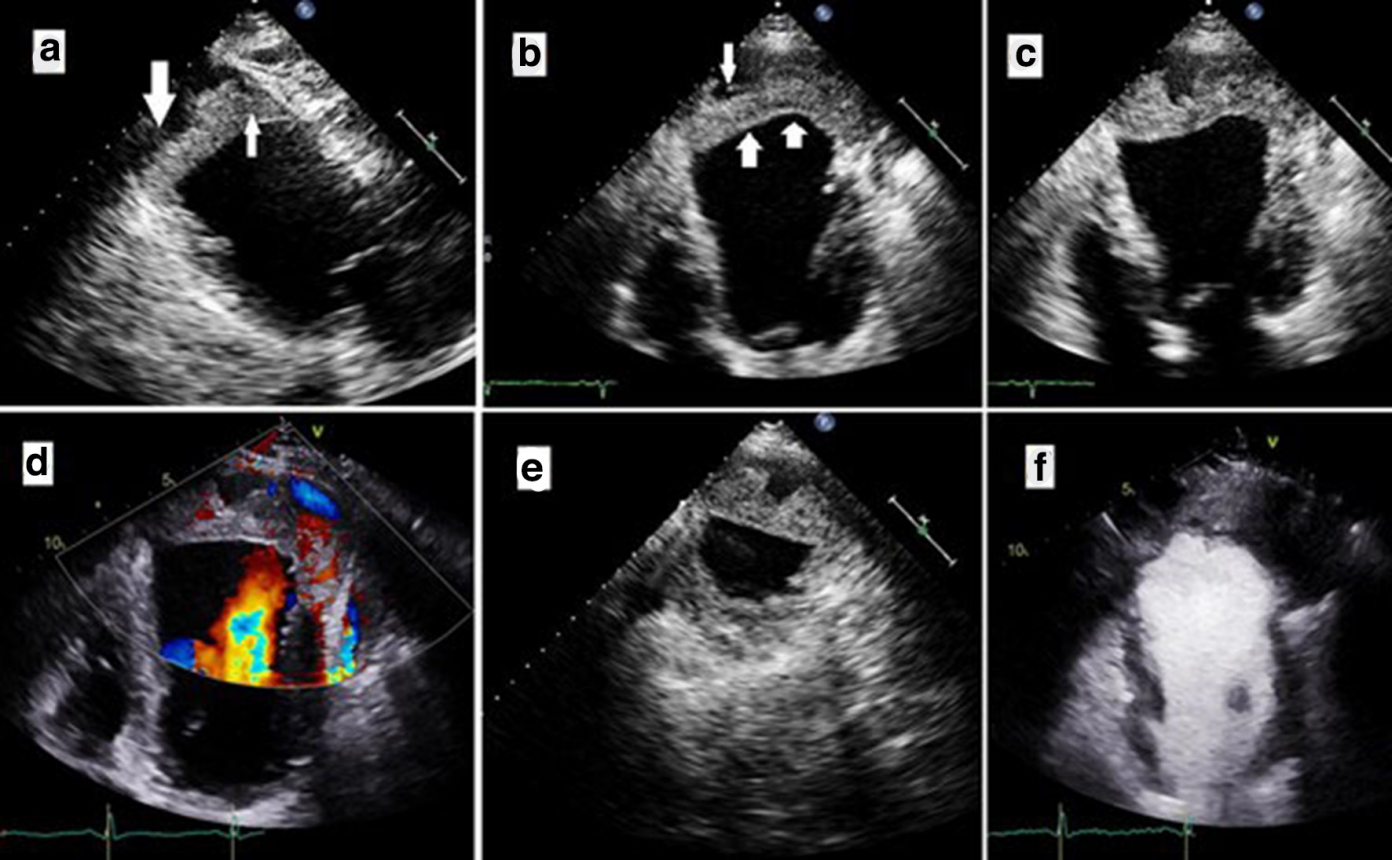
Transthoracic echocardiography: Parasternal long-axis view (a). Apical four-chamber view in systole (b) & diastole (c).Colour Doppler echo (d). Apical parasternal short-axis view (e). Contrast echo (f). LV apical partially thrombosedneocavity (arrows in a, b), the inner boundary of which is made by an endomyocardial layer displaying paradoxical diastolic contraction (c).No detectable communication between the LV cavity and the neocavity neither in the colour Doppler nor the contrast echo images. LV ( left ventricle).

Bedside TTE was performed on the same day of admission and showed (Figure 1) & (Supplementary Videos 1–3) LV apical cavitary lesion with internal heterogeneity (likely to be thrombosed content). The neocavity was separated from the LV cavity by a contracting wall displaying paradoxical diastolic contraction, indicating that it is an endomyocardial layer. There was no detectable communication between the LV cavity and the neocavity in the contrast echocardiography. Doppler images showed no flow inside the neocavity. The estimated LV ejection fraction (EF) was 20–25%. No pericardial effusion was detected. Conventional coronary angiography revealed a severe three-vessel disease.

Supplementary Video 1.

Supplementary Video 2.

Supplementary Video 3.

CMR was performed on the next day of admission on a 1.5 T system (SignaHDx Series, GE Healthcare, Chicago, IL). Retrospectively gated breath-hold steady-state free precession sequences (SSFP-FIESTA) were obtained in the four-chamber and two-chamber long axes, then in the short axis slabs from the LV base to the apex and axial chest slabs covering the heart. T_1_ weighted double inversion recovery and T_2_ weighted double inversion recovery with fat suppression were applied in the axial chest plane. Contrast MRA (magnetic resonance angiography) was performed with hree-dimensional reformates then late gadolinium enhancement (LGE) images were acquired in the short axis, four-chamber, and two-chamber planes.

**Figure 2.**
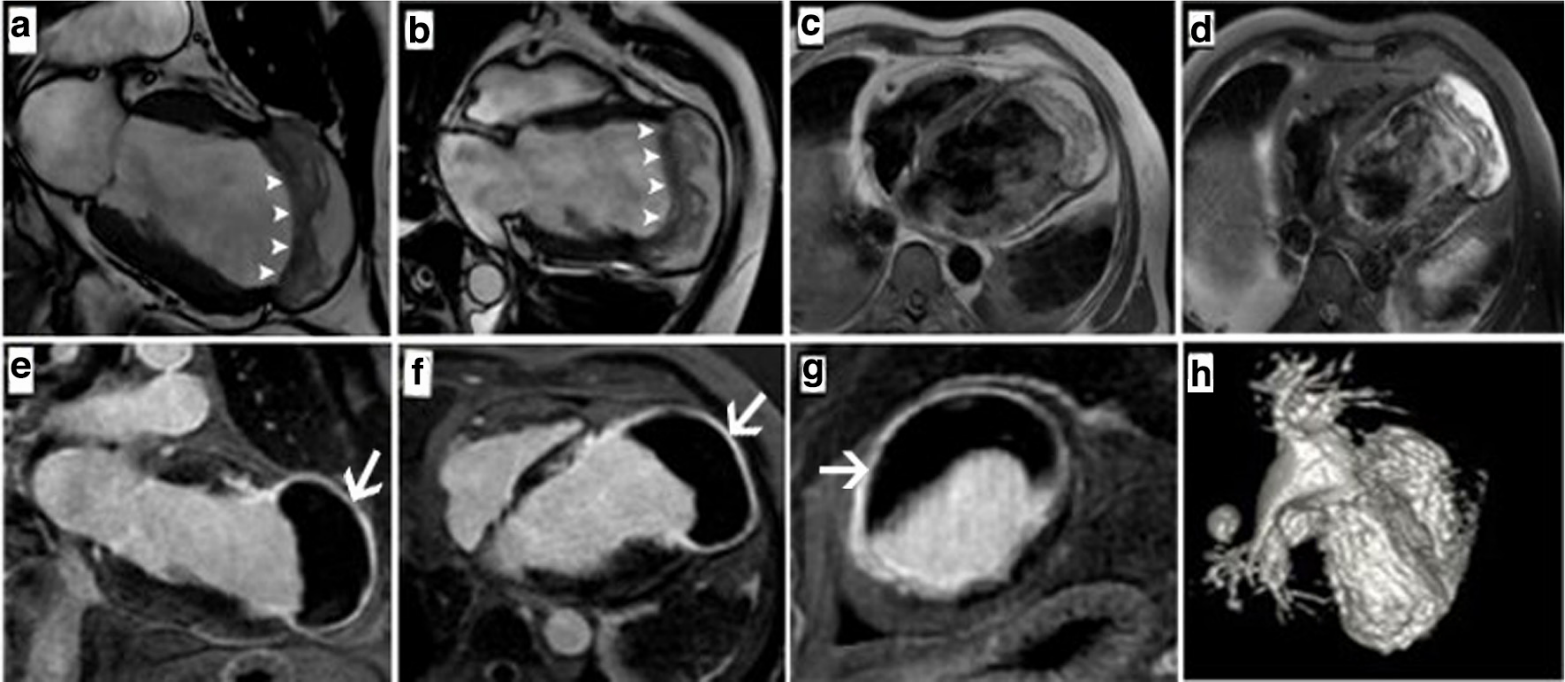
Cardiovascular magnetic resonance: Two-chamber and four-chamber balanced steady-state gradient echo cine CMR (a, b). Axial chest T_1_W-DIR (c). Axial chest T_2_W-DIR-FS (d). Two-chamber, four-chamber, and short-axis LGE (e, f & g). Heart 3D-contrast-enhanced MRA. The LV IMDH appears as a large contained partially thrombosed cystic lesion displaying high blood signal intensity in T_1_W-DIR and T_2_W-DIR. The cyst is delineated by an endomyocardial layer toward the LV cavity (arrowheads in a, b), and by an enhanced outer myocardial layer toward the epicardial surface (arrows in e, f & g). No detectable contrast leakage into the cyst from the LV cavity in post-contrast MRA (H). 3D( three-dimensional); IMDH ( intramyocardial dissecting hematoma); LGE ( late gadolinium enhancement); MRA (magnetic resonance angiography); IMDH (intramyocardial dissecting hematoma); T_1_W-DIR *(* T_1_ weighted double inversion recovery); T_2_W-DIR-FS (T_2_ weighted double inversion recovery with fat suppression).

CMR images are presented in (Figure 2) & (Supplementary Videos 4 and 5). CMR showed enhanced infarcted five LV segments in LAD (left anterior descending coronary artery) territory (mid-anterior, mid-anteroseptal, apical anterior, apical septal, and apex segments) with a large intramyocardial dissecting cystic lesion delineated by endomyocardial flap toward the LV cavity (clearly appreciated on FIESTA images) and by the enhanced infarcted myocardium toward the pericardial space. The dissecting hematoma displayed heterogeneous signal intensity, the thrombus that was of isointense signal to the myocardium, and high signal intensity to the myocardium of the blood products in T_1_ weighted and T_2_ weighted images. On the LGE sequence, the cystic lesion displayed homogenous signal void signal intensity outlined by the enhanced myocardium toward the pericardium. There was no detectable endomyocardial tear. Contrast-enhanced MRA showed no contrast leakage from the LV cavity into the hematoma. A rim of pericardial effusion was noted. The patient was treated conservatively according to the consensus of the heart team, including cardiologists, cardiovascular surgeons, interventional cardiologists, and anaesthesiologist. They went with the conservative approach due to the high risk of surgery, the large size and wide extension of the IMDH, and the poor LV function.

Supplementary Video 4.

Supplementary Video 5.

The patient was treated medically with anti-failure medications. Anticoagulants were not used for fear of increasing the size of the hematoma. Follow-up TTE after 4 months showed no significant change as regards the size and the echo pattern of the hematoma in the last TTE before discharge. The patient was haemodynamically stable.

### Discussion

The worldwide incidence of IMDH has not been adequately reported. It is an uncommon but under recognised complication of myocardial infarction.^5^ LV free wall rupture is the third most common cause of death after acute myocardial infarction, and immediate surgical repair is the treatment of choice. In such cases, a tear from the endocardium through the myocardium and epicardium leads to cardiac tamponade, imminent left ventricle failure, and death.^6^ If the tear is confined to the myocardium, an intramyocardial hematoma (IMH) can be formed as a neocavitation enclosed by the myocardium. The hematoma was thought to be due to the rupture of the intramyocardial vessels, diminished tensile strength of the infarcted myocardium, or due to sudden increased perfusion pressure.^7^

TTE is the most commonly used first-time imaging modality, but CT or CMR is advised if the patient is clinically stable.^8^ Some previous variable publications suggested the establishment of IMDH diagnosis echocardiographically if the patient had at least three of the following supporting findings:(1) formation of a neocavitation with central echolucency (2) mobile contracting endomyocardial border (3) the neocavity is outlined by the myocardium from outside (4) continuity between the ventricular cavity and the dissecting hematoma (5) detection of Doppler coloured flow within the cavity (6) changing of the echo pattern of the neocavity on follow-up (7) partial or complete absorption of the cystic cavity.^1,8^

CMR is the only imaging modality that can detect precisely IMH owing to the characteristic signal intensity of the blood products. However, the optimal CMR sequences to identify IMDH are still a matter of debate. Kumar et al, in his paper, concluded that T2*-CMR could adequately quantify the myocardium reperfusion haemorrhage.^9^ Kali et al reported that T2* is more suitable than T_2_ weighted CMR in the detection of the acute IMH.^10^ While Pedersen et al study concluded that T_1_ weighted inversion recovery sequences (T_1_WIR) have high sensitivity and specificity in the detection of experimental IMH in porcine myocardium as compared to T_2_ short tau inversion recovery and T_2_*-weighted sequences.^11^ In our case, the IMDH was well-depicted in the cine FIESTA, and in the DIR (both T_1_- and T_2_ weighted). In the conventional LGE, the enhanced infarcted inner endomyocardial layer could not be differentiated from the adjacent bright LV blood. So, we recommend using the novel dark-blood LGE method to nullify the LV blood magnetisation for better detection of the ischaemic myocardial scar in such similar cases. It had been published that dark blood LGE imaging improved the depiction of myocardial fibrosis in the cases with low contrast with the contiguous blood pool.^12,13^

The management of patients with IMDH, either via surgical or conservative approach, depends on various factors: the haemodynamic stability status of the patient, the size of the dissecting hematoma, LV function, and the experience of each surgical centre.^5,10^ Conservative management is a suitable viable approach for patients with haemodynamic stability, large-sized dissecting area, or those with reduced LV function and dilated cardiomyopathy.^14^ Till now, the short-to-midterm prognosis of IMDH in patients who were not treated surgically is poor. However, many IMDH cases treated conservatively were published showed a fair mid-to-long-term survival.^14,15^

Herein, we represent a rare case of a large LV partially thrombosed IMDH. The lesion is suspected by TTE and very well characterised in CMR.

## Learning points

- Intramyocardial dissecting hematoma is an uncommon and under recognised complication of myocardial infarction.
- Echocardiography and CMR deliver an accurate diagnosis of IMDH.
- Conservative management is a viable approach for IMDH patients who are haemodynamically stable, with a large-sized IMDH, or those with impaired LV function.
